# Supplementary material for: Vitamin D Modified DSS-Induced Colitis in Mice via STING Signaling Pathway
Source: Biology (Basel). 2025 Jun 18;14(6):715. doi: 10.3390/biology14060715 (PMC12190092; doi:10.3390/biology14060715)
Supplement: Supplementary file 1 [file biology-14-00715-s001.zip › Supplementary Materials S1: 16s-report of gut microbiota/Groups/diff_analysis/UnivarTestSpecies/figure/boxplot/top10.pdf]

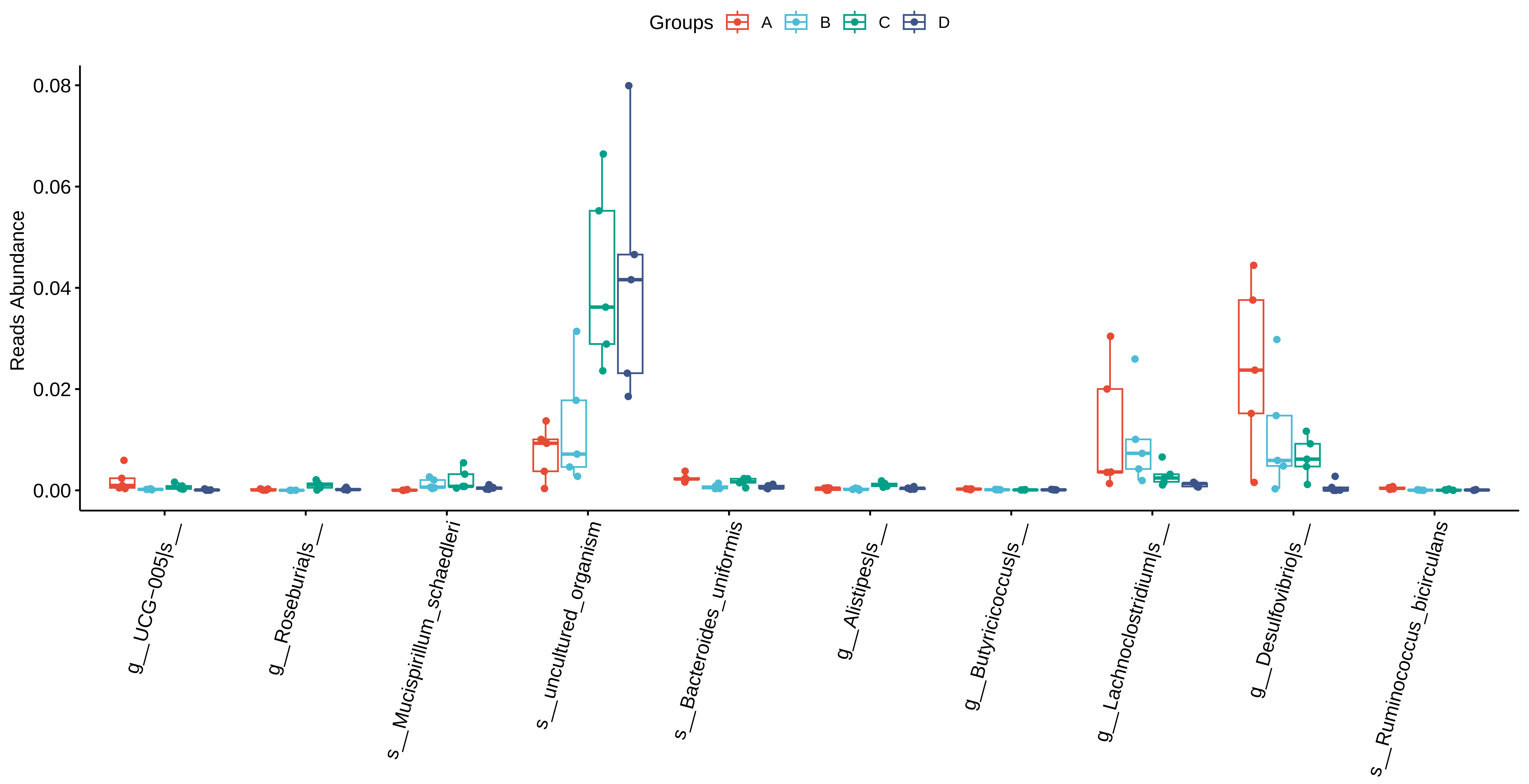

Groups
A
B
C
D
Reads Abundance
0.08
0.06
0.04
0.02
0.00
g__UCG-005|s__
g__Roseburia|s__
s__Mucispirillum_schaedleri
s__uncultured_organism
s__Bacteroides_uniformis
g__Alistipes|s__
g__Butyricicoccus|s__
g__Lachnoclostridium|s__
g__Desulfovibrio|s__
s__Ruminococcus_bicirculans
